# Improving Electronic Health Record (EHR) data linkages for identifying Aged Care Residents through publicly available data

**DOI:** 10.23889/ijpds.v9i5.2767

**Published:** 2024-09-18

**Authors:** Tanya Ravipati, Richard Beare, Velandai Srikanth, Alison Carver, Lei Huang, Rashwinder Dhillon, Nadine E Andrew

**Affiliations:** 1 National Centre for Healthy Ageing; 2 Peninsula Clinical School

## Introduction

Accurate identification of Aged Care residents accessing hospital services is challenging due to multiple hospital Electronic Health Record (EHR) systems and entry points resulting in inconsistent addresses and coding. Improving accuracy and completeness of addresses is crucial for cohort identification and data linkage.

## Objective

Develop methods to validate cross-sectoral data linkages to improve identification of aged care residents within EHR systems.

## Approach

Among individuals aged ≥65 years (or ≥50 identifying as Aboriginal or Torres Strait Islander) between January 2013 to December 2023 within our Data Platform, we first used data items mandated by the Department of Health to identify aged care residency. Data quality was enhanced using a residential address index derived from Australian Geocoded National Address File (G-NAF). We evaluated sensitivity, specificity, and Positive Predictive Values (PPVs) of recorded addresses against the Australian Institute of Health and Welfare (AIHW) aged care services list.

## Results

Among 409,677 addresses extracted, 55.4% (raw) had sufficient data for validation. This increased to 89.9% (enhanced) with G-NAF. Among 79,028 admitted and 88,876 emergency patients, 22.7% and 18.4% respectively were aged care residents. We compared performance of raw vs enhanced comparisons were: sensitivity (admission: 34.1% vs 73.8%), (emergency: 42.6% vs 66.2%); specificity (admission: 96.2% vs 90.9%), emergency (96.8% vs 95.2%); Positive Predictive Value (admission: 61.3% vs 58.8%), (emergency:  64.2% vs 65.3%).

## Conclusion

Our approach significantly increased sensitivity without sacrificing specificity, enhancing Aged Care resident identification in EHR data. It also improved linkage with external datasets and Statistical Area Level 1 (SA1) mapping.

